# Decision making under uncertainty

**DOI:** 10.3389/fnins.2013.00218

**Published:** 2013-11-20

**Authors:** Kerstin Preuschoff, Peter N. C. Mohr, Ming Hsu

**Affiliations:** ^1^Laboratory of Computational Neuroscience, École Polytechnique Fédérale de LausanneLausanne, Switzerland; ^2^Laboratoire de Recherché en Neuroimagerie, Le Centre Hospitalier Universitaire VaudoisLausanne, Switzerland; ^3^Information Processing and Economic Decision Making, Department of Psychology, Universität KonstanzKonstanz, Germany; ^4^Economic Psychology, Department of Psychology, University of BaselBasel, Switzerland; ^5^Psychology of Emotions and Affective Neuroscience, Department of Educational Science and Psychology, Freie Universität BerlinBerlin, Germany; ^6^Neuroeconomics Laboratory, Haas School of Business, University of California BerkeleyBerkeley, CA, USA; ^7^Helen Wills Neuroscience Institute, University of California BerkeleyBerkeley, CA, USA

**Keywords:** decision making, decision neuroscience, neuroeconomics, uncertainty, risk, contextual influences, situational influences, individual differences

In our everyday life we often have to make decisions with uncertain consequences, for instance in the context of investment decisions. To successfully cope with these situations, the nervous system has to be able to estimate, represent, and eventually resolve uncertainty at various levels. That is, not only are there different forms of uncertainty with different consequences for behavior and learning but research indicates that the processing of uncertainty highly depends on situation and context. The present research topic includes both review and original research articles that seek to shed light on the neural processes underlying decision making under uncertainty with a particular focus on situational and contextual influences.

First, Bland and Schaefer (2012) review the diverse (and often overlapping) definitions of uncertainty. They identify three main forms—expected uncertainty (including risk), unexpected uncertainty and volatility—and review theoretical and empirical evidence that supports this dissociation. Several original research articles then aim to either directly compare different forms of uncertainty or to identify further dissociations within these forms. Payzan-LeNestour and Bossaerts (2012) systematically vary unexpected and estimation uncertainty to study what drives exploration (as opposed to exploitation). They report that humans both seek out new reward opportunities (“curiosity motive”) and avoid the unknown (“cautiousness motive”), resulting in exploration and exploitation, respectively. O'Reilly (2013) addresses the same forms of uncertainty in the context of learning with a particular focus on how an organism should adapt their rate of learning in changing environments. Hansen et al. (2012) on the other hand show that decisions made under perceptual vs. categorical uncertainty are differentially affected by prior knowledge such that prior knowledge increases visual cortical activity when uncertainty is driven by the sensory stimulus itself rather than at the cognitive level.

The next set of papers explores situational and contextual aspects of expected uncertainty. First, Studer et al. (2012) demonstrate that neural responses in a distributed network of choice under risk increase when subjects actively choose a risky gamble as opposed to being passively exposed to risk when a computer chooses that gamble. Kim et al. (2012) study what information decision makers attend to when either choosing between two lotteries or betting on a single lottery. Using eye-tracking data they observe task-dependent attentional shifts from probabilities to amounts which may influence the (neural) computation of value. Consequently, individuals often chose options with higher probabilities but place higher bids on options with higher amounts. Schönberg et al. (2012) used the Balloon Analog Risk Taking task to study the neural network underlying naturalistic risk-taking. They find that brain activity in a network previously related to risk increases as individuals continue to inflate a balloon—thus, increasing their risk—while activity in a value-related brain region decreases at the same time. Levin et al. (2012) then review the literature on how risk processing differs between the gain and loss domain. They argue that different neural systems indicate different neural and psychological processes for risk-taking in gains and losses. Finally, Heilbronner and Hayden (2013) round off this set of papers by providing an account of risk-seeking behavior. While risk-seeking is usually observed in only a minority of human study participants, it is the dominant form of risk preference observed in monkey studies. Heilbronner and Hayden review the literature on this phenomenon and argue that monkeys aren't risk-seeking *per se* but are driven toward risk-seeking by experimental design and training and that under similar conditions rats and humans would behave the same way.

Finally, a third set of papers represents an increasingly fertile area of research by connecting risk-taking to the social contexts and affective processes underlying behavior. Tang et al. (2011) report that socially anxious individuals demonstrate decreased risk aversion and that the degree of social anxiety correlates with activity in anterior insula. Jung et al. (2013) compare the number of risky choices participants made for themselves or for others. They find that at low probabilities subjects are less risk taking for own decisions as opposed to high probabilities where the effect is reversed. This difference in preferences toward risk is underlined by partially distinct neural networks that are recruited when choosing for oneself or for others. Using a model-based approach, Zhu et al. (2012) connect social risk and learning, and demonstrate that age-related differences in social learning can be succinctly captured by a set of models widely used in economics. Gaertig et al. (2012) use an ultimatum game to show that positive social information about the proposer increases acceptance rates by the responder. This effect was further enhanced by the presence of uncertainty. Finally, Wu et al. (2012) provide an affective neuroscience account of decision making under risk thereby connecting the quantitative approach of economic and financial theories with the psychological approach which focuses on emotion and cognition.

In sum, the papers presented in this research topic demonstrate several points: First, to fully understand decision making under uncertainty one has to first dissociate different forms of uncertainty. Each form impacts behavior and learning in a different way (Figure 1). Second, choices under each form of uncertainty can itself be impacted by situational and contextual factors. Third, social context is an important source of uncertainty that is often driven or influenced by affective processes. We can further contend that risk remains the most popular and most powerful form of uncertainty for studying choice under uncertainty. The quantitative framework provided by choice under risk allows the careful study of the impact of situational and contextual factors on preferences and choice. However, as most situations in real life are infused with unexpected uncertainty and volatility rather than expected uncertainty (risk), future research will show how the factors identified in this issue influence other forms of uncertainty, to which degree common mechanism exist and how they can account for the various influences identified so far.

**Figure 1:**
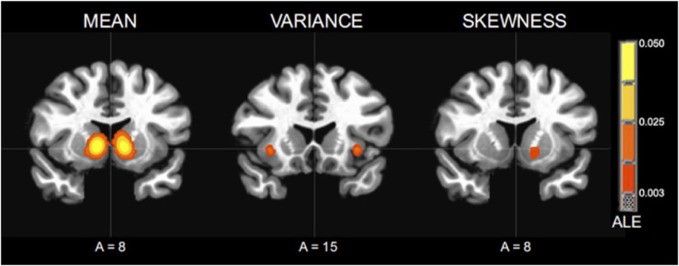
**Activation Likelihood Estimate (ALE) meta-analytic maps for high versus low mean, variance, and skewness**. ALE of mean: bilateral NAcc. ALE of variance: bilateral anterior insula. ALE of skewness: left NAcc. (based on Wu et al., 2012).

## References

[B1] Bland A. R. Schaefer A. (2012). Different varieties of uncertainty in human decision-making. Front. Neurosci. 6:85 10.3389/fnins.2012.00085 22701401 PMC3370661

[B2] Gaertig C. Moser A. Alguacil S. Ruz M. (2012). Social information and economic decision-making in the ultimatum game. Front. Neurosci. 6:103 10.3389/fnins.2012.00103 22783164 PMC3390557

[B3] Hansen K. A. Hillenbrand S. F. Ungerleider L. G. (2012). Effects of prior knowledge on decisions made under perceptual vs. categorical uncertainty. Front. Neurosci. 6:163 10.3389/fnins.2012.00163 23162424 PMC3499736

[B4] Heilbronner S. R. Hayden B. Y. (2013). Contextual factors explain risk-seeking preferences in rhesus monkeys. Front. Neurosci. 7:7 10.3389/fnins.2013.00007 23378827 PMC3561601

[B5] Jung D. Sul S. Kim H. (2013). Dissociable neural processes underlying risky decisions for self versus other. Front. Neurosci. 7:15 10.3389/fnins.2013.00015 23519016 PMC3602668

[B6] Kim B. E. Seligman D. Kable J. W. (2012). Preference reversals in decision making under risk are accompanied by changes in attention to different attributes. Front. Neurosci. 6:109 10.3389/fnins.2012.00109 22833715 PMC3400145

[B7] Levin I. P. Xue G. Weller J. A. Reimann M. Lauriola M. Bechara A. (2012). A neuropsychological approach to understanding risk-taking for potential gains and losses. Front. Neurosci. 6:15 10.3389/fnins.2012.00015 22347161 PMC3273874

[B8] O'Reilly J. X. (2013). Making predictions in a changing world-inference, uncertainty, and learning. Front. Neurosci. 7:105 10.3389/fnins.2013.00105 23785310 PMC3682109

[B9] Payzan-LeNestour E. Bossaerts P. (2012). Do not bet on the unknown versus try to find out more: estimation uncertainty and “unexpected uncertainty” both modulate exploration. Front. Neurosci. 6:150 10.3389/fnins.2012.00150 23087606 PMC3472893

[B10] Schönberg T. Fox C. R. Mumford J. A. Congdon E. Trepel C. Poldrack R. A. (2012). Decreasing ventromedial prefrontal cortex activity during sequential risk-taking: an FMRI investigation of the balloon analog risk task. Front. Neurosci. 6:80 10.3389/fnins.2012.00080 22675289 PMC3366349

[B11] Studer B. Apergis-Schoute A. M. Robbins T. W. Clark L. (2012). What are the odds? The neural correlates of active choice during gambling. Front. Neurosci. 6:46 10.3389/fnins.2012.00046 22529770 PMC3328778

[B12] Tang G. S. van den Bos W. Andrade E. B. McClure S. M. (2011). Social anxiety modulates risk sensitivity through activity in the anterior insula. Front. Neurosci. 5:142 10.3389/fnins.2011.00142 22319462 PMC3250055

[B13] Wu C. C. Sacchet M. D. Knutson B. (2012). Toward an affective neuroscience account of financial risk taking. Front. Neurosci. 6:159 10.3389/fnins.2012.00159 23129993 PMC3487049

[B14] Zhu L. Walsh D. Hsu M. (2012). Neuroeconomic measures of social decision-making across the lifespan. Front. Neurosci. 6:128 10.3389/fnins.2012.00128 23049494 PMC3448294

